# Co-recognition of β-glucan and chitin and programming of adaptive immunity to *Aspergillus fumigatus*

**DOI:** 10.3389/fmicb.2015.00344

**Published:** 2015-04-21

**Authors:** Nansalmaa Amarsaikhan, Steven P. Templeton

**Affiliations:** Department of Microbiology and Immunology, Indiana University School of Medicine – Terre Haute, Terre Haute, IN, USA

**Keywords:** *Aspergillus fumigatus*, fungal infection, aspergillosis, innate recognition, adaptive immunity, β-glucan, chitin, cell wall modulation

## Abstract

The prevalence of fungal infections has increased concurrently with increases in immune suppressive therapies and susceptible individuals. Opportunistic fungal pathogens such as *Aspergillus fumigatus* may exhibit invasive growth and dissemination resulting in a high mortality rate. Herein, we discuss how immune sensing of germination directs innate immune responses and programs adaptive responses that could promote or impair immune protection during periods of heightened susceptibility. In infected individuals, Th1 responses are the most protective, while Th2 responses lead to poor disease outcomes. In particular, the roles of β-glucan and chitin co-recognition in shaping Th1- and Th2-type immunity to fungal infection are explored. We discuss how fungal responses to environmental stresses could result in decreased immune protection from infection, particularly in response to anti-fungal drugs that target β-glucan synthesis. Furthermore, we consider how experimental modulation of host-pathogen interactions might elucidate the mechanisms of protective and detrimental immunity and the potential of current and future studies to promote the development of improved treatments for patients that respond poorly to existing therapies.

## Introduction

*Aspergillus fumigatus* is an opportunistic fungal pathogen abundant in indoor and outdoor environments, causing fungal infection in immune suppressed individuals and exacerbating chronic pulmonary conditions (Hohl and Feldmesser, 2007; Kwon-Chung and Sugui, 2013). The small size of *A. fumigatus* conidia promotes aerosol formation and thus frequent contact with airways of potential hosts. Small to moderate amounts of conidia are often removed by alveolar macrophages without a significant inflammatory response. When larger numbers of conidia are inhaled, more conidia are allowed to germinate, stimulating in an inflammatory response. Swelling of *A. fumigatus* conidia follows the degradation of the outermost hydrophobic rodlet layer, thus exposing the inner cell wall layer composed of a complex network of immune-stimulating polysaccharides (Thau et al., 1994; Latgé, 1999; Paris et al., 2003; Aimanianda et al., 2009). Since these cell wall components are not found in mammalian hosts, specific host recognition receptors have evolved as a mechanism to signal appropriate early inflammation and the subsequent development of protective innate and adaptive immune responses.

Recent studies have indicated a variety of responses to particulate forms of fungal pathogen-associated molecular patterns (PAMPs). However, in natural *A. fumigatus* exposure and infection, fungal PAMPs are recognized in concert on the germinating conidial surface, and it is thus likely that this combined recognition results in programming of immune profiles not observed in studies of purified, particulate cell wall components. Of the PAMPs contained in the cell wall of *A. fumigatus*, many studies have focused on the covalently linked fibrillary core polysaccharides β1-3-glucan (β-glucan) and chitin; both known to be immune stimulatory in purified, particulate form (Lenardon et al., 2010; Drummond and Brown, 2011). Furthermore, the amorphous cell wall components α1-3-linked glucan (α-glucan), galactomannan, and galactosaminogalactan (GAG) also act to modify immune responses to infection (Bozza et al., 2009; Gravelat et al., 2013; Latge and Beauvais, 2014), though their direct contributions to the development of protective or detrimental immunity are less clear. Recent studies reported that the expression of fungal PAMPs varies considerably depending on available nutrients, temperature, oxygen levels, and the presence of anti-fungal drugs (Verwer et al., 2012; Shepardson et al., 2013; Beauvais et al., 2014). When combined, these and other potential cellular and metabolic stressors may ultimately result in distinct patterns of recognition and cell signaling with the potential to program equally distinct profiles of adaptive immunity. However, our current understanding of these pathways of recognition and their influence on adaptive immunity is basic and preliminary, and more detailed studies of combined recognition of fungal PAMPs during germination are needed. Herein, we examine the role of individual and aggregate pattern recognition in the programming of immunity to *A. fumigatus*, focusing on the immune responses to β-glucan and chitin. We also consider the evidence that fungal cell wall modulation due to environmental stresses like antifungal drug exposure could either enhance or diminish immune protection from infection.

## Early Recognition of Fungal Germination

### β-Glucan/dectin-1

The *A. fumigatus* cell wall consists of covalently bound β-glucan, chitin, galactomannan and α-glucan that are absent in mammals, and thus present prime targets for pattern recognition receptors (PRRs) on host cells (Chai et al., 2011). β-glucan is recognized by the C-type lectin receptor dectin-1 and has been studied extensively with infection models of *A*. *fumigatus* and other pathogenic fungi (Drummond and Brown, 2011). Downstream signaling of dectin-1 activation promotes cellular antifungal responses including phagocytosis, ROS production, and inflammatory cytokine production. Mutations in human dectin-1 rendered individuals more susceptible to invasive aspergillosis, and infected dectin-1-deficient mice displayed increased pathology with decreased neutrophil recruitment and impaired cytokine production (Steele et al., 2005; Gersuk et al., 2006; Cunha et al., 2010; Gessner et al., 2012). The inflammatory response initiated by binding of dectin-1 receptor on resident cells is strengthened when combined with signaling through toll-like receptors (TLR) that are co-expressed within an immunological synapse (Goodridge et al., 2011; Inoue and Shinohara, 2014), resulting in synergistically increased cytokine production and activation of inflammatory signaling pathways (Mambula et al., 2002; Hohl et al., 2005; Gersuk et al., 2006; Dennehy et al., 2008). Thus, dectin-1 and associated PRR recognition provide immune signals essential for protective immunity to *A. fumigatus* infection.

Conidial surface β-glucan is initially recognized by epithelial cells, macrophages and dendritic cells (Drummond and Brown, 2011; Osherov, 2012). Epithelial cells act as the first barrier and immunologically active surface in host tissues, serving as non-professional phagocytes where engulfed conidia persist in the pulmonary epithelial space (Heinekamp et al., 2015). Airway epithelial cells activated a panel of antimicrobial genes in a β-glucan-mediated response to *A. fumigatus* (Evans et al., 2010; Sun et al., 2012), and secreted TNF-α, IL-8 (CXCL-8) and GM-CSF (Sun et al., 2012), indicating an important role for these cells in neutrophil recruitment that is essential for protection from invasive infection (Bonnett et al., 2006; Mircescu et al., 2009). Similar to epithelial cells, alveolar macrophages from dectin-1 knockout mice lacked the ability to produce IL-1α/β, TNF-α, CCL3/4 (MIP-1α/β), and CXCL1 (KC) in response to *A. fumigatus* (Werner et al., 2011). A dectin-1/CARD9 pathway promoted early neutrophil influx, although initial recruitment may be mediated by a hypoxia inducible factor-α/IL1R1/MyD88 pathway (Shepardson et al., 2014; Caffrey et al., 2015; Jhingran et al., 2015). In neutrophils, β-glucan recognition by dectin-1 promoted production of reactive oxygen species (Kennedy et al., 2007). Neutrophils produced dectin-1-mediated IL-17A in the presence myeloid cells in response to *A. fumigatus* (Werner et al., 2011) that likely serves as a feedback signal for increased neutrophil recruitment via stimulation of epithelial cells to produce TNF-α, IL-8, and G-CSF (Iwakura et al., 2011). In addition to neutrophils, NK cells are recruited early after *A. fumigatus* infection by a CCL2-dependent mechanism, and provide protection through IFN-γ secretion and subsequent activation of macrophages (Morrison et al., 2003; Park et al., 2009) and also potentially through enhanced neutrophil killing (Roilides et al., 1993). Inflammatory monocytes also provide protection from invasive infection, similarly in part by enhancing neutrophil conidiacidal activity (Espinosa et al., 2014). In DCs, TLR and dectin-1 signaling mediated β-glucan-induced secretion of TNF-α and IL-12 (Gantner et al., 2003; Mezger et al., 2008). In response to *A. fumigatus* conidia, Dectin-1 also promoted early lung protection and fungal allergy via secretion of IL-22, a cytokine important in activation of antimicrobial effectors at mucosal surfaces (Gessner et al., 2012; Lilly et al., 2012). Thus, dectin-1 recognition of β-glucan exposure in *A. fumigatus* results in the activation of an array of inflammatory cytokines and chemokines that promote early protection from infection.

### Chitin

Chitin is a fungal cell wall polysaccharide that is abundant in parasites, insects, and crustaceans (Da Silva et al., 2010; Lenardon et al., 2010; Muzzarelli, 2010). Chitin microfibrils, covalently linked with β-glucan, impart a strong rigidity to the cell wall of fungal hyphae. The results of several studies examining immune responses to purified chitin indicate that particle size, concentration, and degree of acetylation are important determinants of cytokine profiles and inflammatory cell recruitment (Shibata et al., 1997; Da Silva et al., 2009; Wagener et al., 2014). Low concentrations of chitin particles between 1 and 10 μm induced macrophage IL-10 secretion, while increased concentrations resulted in increased TNF secretion. In contrast, larger chitin particles (50–100 μm) promoted lung eosinophilia and alternative macrophage activation (Reese et al., 2007; Kogiso et al., 2011; Roy et al., 2012). Chitin exposure increased expression of lung epithelial CCL2, IL-25, IL-33, and TLSP that mediated recruitment of eosinophils and promoted M2 (alternatively activated) macrophage activation (Islam and Luster, 2012; Roy et al., 2012; Van Dyken et al., 2014). Furthermore, chitin-induced IL-25, IL-33, and TLSP induced type 2 innate lymphoid cells (ILC2) to secrete IL-5 and IL-13, cytokines essential for eosinophil recruitment and M2 macrophage activation (Van Dyken et al., 2014). In addition to purified particles, inhaled fungal chitin from house dust, hyphal extracts, and conidia also promoted lung eosinophil recruitment in mice that was decreased in the presence of constitutively expressed acidic mammalian chitinase (AMCase; Van Dyken et al., 2011; O’Dea et al., 2014). These studies demonstrate that innate recognition of purified or fungal chitin induces recruitment of eosinophils and promotes M2 macrophage activation.

In contrast to β-glucan, a distinct chitin recognition receptor has not been fully characterized. To date, the only chitin-specific receptor identified is FIBCD1, a type II transmembrane protein apically expressed in gut tissues (Schlosser et al., 2009). However, several PRRs specific for other microbial PAMPs are associated with chitin-mediated responses. Chitin-induced macrophage secretion of IL-17A and TNF-α were dependent on the TLR-2/MyD88 pathway and dectin-1/TLR2 expression, respectively (Da Silva et al., 2008, 2009). IL-10 secretion in response to smaller chitin particles was dependent on mannose receptor, NOD2 and TLR9 (Wagener et al., 2014). In addition, the cytosolic C-type lectin RegIIIγ also binds chitin (Cash et al., 2006). Notably, the well-described ligand shared by TLR2, NOD2, and RegIIIγ is peptidoglycan, an essential cell wall component of gram-positive bacteria that, like chitin, consists of a carbohydrate backbone containing *N*-acetylglucosamine residues (GLcNAc). Furthermore, RegIIIγ and other C-type lectins that bind GlcNAc containing polysaccharides also bind mannan (Drickamer, 1992; Cash et al., 2006). It is possible that this structural similarity enables mannose receptor-mediated responses to chitin particles. The innate immune signals involved in chitin recognition are nonetheless complex, and future studies are needed to determine the importance of each of these recognition molecules in innate immune responses to chitin-containing pathogens.

## Innate Immune Effectors Program Adaptive Immunity

Although alveolar macrophages and neutrophils are critical for killing dormant or germinating conidia and hyphae, monocytes, NK cells, NKT cells, plasmacytoid DCs, and eosinophils may also provide early protection from infection (Morrison et al., 2003; Mircescu et al., 2009; Cohen et al., 2011; Ramirez-Ortiz et al., 2011; Espinosa et al., 2014; Lilly et al., 2014). Furthermore, cytokines produced by these cells are involved in the programming of protective or non-protective adaptive immune responses. In particular, CD4 (T-helper) and CD8 (cytotoxic) T cells provide significant protection from *A. fumigatus* infection and are therefore considered important targets for vaccination studies (Cenci et al., 2000; Perruccio et al., 2005; Chai et al., 2010; Romani, 2011; Carvalho et al., 2012). However, Th1 responses are the most protective, while Th2 responses result in poor disease outcomes. The level of protection conferred by Th17 cells and IL-17 is not clear, as conflicting studies reported impaired or enhanced early protection after antibody depletion of IL-17A (Zelante et al., 2007; Werner et al., 2009). In a model of fungal keratitis, IL-17A was protective, although the cellular sources of IL-17A attributed to this protection included neutrophils in addition to Th17 cells (Taylor et al., 2014). In addition to neutrophils, γδ T cells may also be an important source of IL-17A, particularly in the lung, although their role in protection is unclear and may be subset-dependent (Roark et al., 2008; Romani et al., 2008). NK and invariant NKT cells may be early sources of IFN-γ during infection (Bouzani et al., 2011; Cohen et al., 2011), while basophils or NKT cells may provide innate production of IL-4 in the development of allergy/Th2 responses (Taniguchi et al., 2003; Liang et al., 2012). Therefore, in addition to proinflammatory cytokines produced by innate cells, early production of T helper cytokines provides an early window into the subsequent development of protective or detrimental adaptive responses.

Perhaps the most consequential cell in initiating adaptive immunity to fungal infection is the DC (Romani, 2011; Wuthrich et al., 2012). Initiation of a protective adaptive immune response against *A. fumigatus* is partly dependent on the actions of DCs stimulated through activation of fungal PRRs. Monocytes recruited into the lung shortly after *A. fumigatus* infection differentiated into DCs that were critical for induction of Th1 responses that are increased in the absence of dectin-1 (Hohl et al., 2009; Rivera et al., 2011). Rather than promote Th1 responses, dectin-1 recognition induced Th17 responses to *A. fumigatus* (Werner et al., 2009; Rivera et al., 2011). Accordingly, direct stimulation of DCs with purified β-glucan stimulated TNFα, yet inhibited TLR-mediated induction of IL-12 (Huang et al., 2009). DC priming of Th2 responses was promoted by the epithelial cytokines TSLP and IL-33 that were also induced in epithelial cells by chitin stimulation (Paul and Zhu, 2010; Van Dyken et al., 2014). Chitin particles also induced generation of C3a in the lungs of mice that is required for DC stimulation of Th2 responses to *Aspergillus fumigatus* hyphal extracts (Roy et al., 2013). DCs thus respond to different fungal PAMPs with distinct cytokine profiles and differentially prime Th responses.

## Co-recognition of β-glucan and Chitin and Programming of Adaptive Immunity

Although many studies have focused on responses to purified fungal PAMPs, actual responses to viable *A. fumigatus* are programmed as a result of co-recognition of multiple PAMPs by multiple PRRs after these ligands are revealed on the surface of germinating conidia. Furthermore, since soluble forms of these ligands are often inhibitory, it has been hypothesized that long fibrillar polysaccharide fungal PAMPs are able to bind to multiple PRRs, thus increasing activation signals in PRR-expressing cells (Latge, 2010). Recognition by multiple PRRs would also be facilitated by clustering formations within the immunological synapse (Goodridge et al., 2011; Inoue and Shinohara, 2014). Results of studies that examined the effects of co-recognition of multiple PAMPs with mixtures of particles or in response to intact conidia provide a contrast to studies focused solely on responses to purified particles. For example, covalently-linked chitin-β-glucan particles induced neutrophil and eosinophil recruitment as well increased chitinase activity, TNF-α and TSLP production in mouse lungs (Dubey et al., 2014). Furthermore, multiple aspirations of viable *A. fumigatus* conidia activated Th1, Th2, and Th17 responses, and the relative expansion of these subsets may depend on the dose, frequency of aspirations, and strain characteristics of the conidia used (Fei et al., 2011; Murdock et al., 2011; O’Dea et al., 2014). Our laboratory identified an isolate of *A. fumigatus* (Af5517) that expressed increased levels of chitin and induced Th2-skewed immunity in the lungs of mice after repeated conidial aspiration (O’Dea et al., 2014). However, an isolate that we identified as relatively low chitin-expressing (Af13073) induced allergic sensitization when the frequency of aspiration was increased and the interval between aspirations was decreased (Fei et al., 2011; Lilly et al., 2012; Amarsaikhan et al., 2014). Interestingly, dectin-1–/– mice displayed increased eosinophil recruitment in response to single or multiple aspirations of *Aspergillus* conidia (Werner et al., 2011; Mintz-Cole et al., 2012). Although not discussed in either report, it is possible that co-recognition of β-glucan by dectin-1 may inhibit signals generated by chitin recognition and early programming of Th2 responses. However, this effect may be overcome by allergic sensitization, as dectin-1 deficient mice did not exhibit increased lung eosinophilia in a model of fungal asthma (Lilly et al., 2012). This is not surprising, considering other differences between models of exposure and sensitization. For example, chitinase expression promotes allergic inflammation in models of allergic sensitization, while in the absence of sensitization chitinase expression decreases eosinophil recruitment in response to chitin particles, fungal extracts, or conidia (Zhu et al., 2004; Reese et al., 2007; Van Dyken et al., 2011; O’Dea et al., 2014). Taken together, these results suggest that in the absence of sensitization, co-recognition of chitin and β-glucan may provide antagonistic signals that result in differential programming of adaptive immunity to *A. fumigatus*.

## Fungal Stress, Cell Wall Modulation, and Consequences for Treatment of Infection

The clinical relevance of cell wall modulation is an important area of current and future investigation. Several lines of evidence suggest that stresses encountered by pathogenic fungi during infection alter the metabolism and cell wall architecture, and thus modulate immune responses toward non-protective programs of adaptive immunity. In the case of *A. fumigatus* and other fungal infection, Th2 immune responses inhibit protective immunity (Cenci et al., 1998, 1999, 2000; Wuthrich et al., 2012). Eosinophils may be partly responsible for this impairment, as Th2-responding mice that lacked eosinophils increased fungal clearance, although the mechanism for this inhibition remains unknown (O’Dea et al., 2014). Numerous reports have demonstrated alteration of fungal cell wall architecture in response to changes in growth conditions or environmental stresses encountered during infection, in both *A. fumigatus* and *Candida albicans* (Ene et al., 2012; Shepardson et al., 2013; Beauvais et al., 2014). *A. fumigatus* growth under hypoxic conditions resulted in increased cell wall β-glucan and chitin that stimulated increased macrophage and neutrophil activation (Shepardson et al., 2013). More importantly, classes of antifungal drugs that directly target the synthesis of cell wall chitin and β-glucan modulate cell wall architecture over the course of infection, and these changes may concomitantly affect host pattern recognition and pathogen clearance. For example, echinocandins directly target the synthesis of β-glucan, while nikkomycins target chitin synthesis (Ostrosky-Zeichner et al., 2010). Moreover, growth of *A. fumigatus* in the presence of the echinocandin caspofungin resulted in increased cell wall chitin, while growth on nikkomycin Z increased β-glucan (Verwer et al., 2012). In a mouse model of *C. albicans* infection, increased cell wall chitin induced by caspofungin treatment mediated echinocandin resistance (Lee et al., 2012). Thus, cell wall modulation in response to the stresses of infection may influence the development of protective immunity and the efficacy of antifungal drug treatment.

## Summary/Conclusion

**FIGURE 1:**
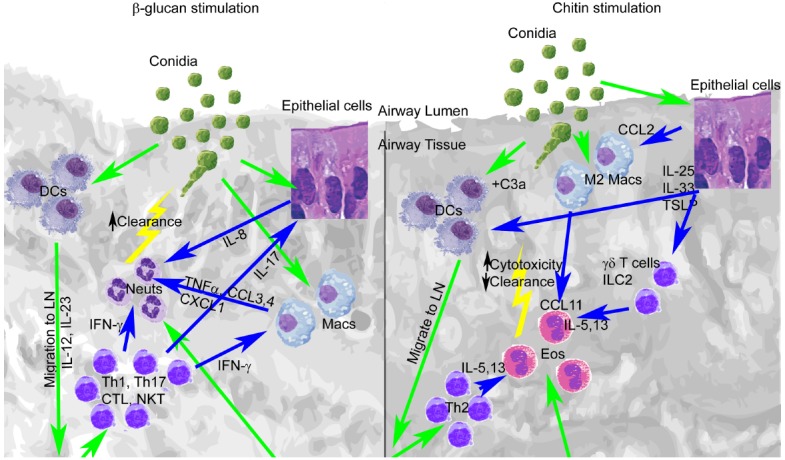
**Early lung recognition of β-glucan and chitin programs distinct profiles of cytokine secretion, leukocyte recruitment, and adaptive immunity.** Left, β-glucan stimulation. Right, chitin stimulation. Recognition of germinating conidia or migration of cells is displayed with green arrows, while cytokine stimulation is shown with blue arrows.

Among the cell wall components of *A. fumigatus*, chitin and β-glucan may stimulate protective or detrimental immune responses, depending on their level of expression and recognition. Other cell wall components such as α-glucan, galactomannan, and GAG may also promote or inhibit the development of protective immunity, although their roles are less understood, and thus require further examination. Early cellular and cytokine signals induced by innate recognition of covalently linked β-glucan and chitin initiate Th1/Th17 or Th2 responses that may alter the balance between protective immunity and damaging inflammation (Figure 1). This co-recognition may be altered by pathogen mutation or in response to environmental stresses encountered during infection, particularly by exposure to antifungal drugs that directly target β-glucan or chitin synthesis. However, the consequences of changes in this recognition to protection from infection are not well understood. Future studies will be required to more completely define the development of protective immunity at the level of host-pathogen interaction, with the goal of introducing and validating new therapies that promote protection and/or target detrimental inflammatory processes that arise within the spectrum of *A. fumigatus*-associated disease.

### Conflict of Interest Statement

The authors declare that the research was conducted in the absence of any commercial or financial relationships that could be construed as a potential conflict of interest.
